# Prevalence of Neutralizing Autoantibodies Against Type I Interferon in a Multicenter Cohort of Severe or Critical COVID-19 Cases in Shanghai

**DOI:** 10.1007/s10875-024-01683-z

**Published:** 2024-03-10

**Authors:** Dongling Shi, Jie Chen, Meng Zhao, Yuanjia Tang, Chen Zhao, Yinpeng Jin, Di Tian, Yixin Liao, Xuebi Wang, Wei Wang, Xiaohong Fan, Zhigang Yi, Xiaohua Chen, Yun Ling

**Affiliations:** 1 Department of Infectious Diseases, Shanghai Public Health Clinical Center, Fudan University, Shanghai, China; 2 Scientific Research Center, Shanghai Public Health Clinical Center, Fudan University, Shanghai, China; 3 Department of Infectious Diseases, Shanghai Sixth People’s Hospital Affiliated to Shanghai Jiao Tong University School of Medicine, Shanghai, China; 4 Key Laboratory of Medical Molecular Virology (MOE/NHC/CAMS), School of Basic Medical Sciences, and Shanghai Institute of Infectious Disease and Biosecurity, Fudan University, Shanghai, China; 5 Shanghai Institute of Rheumatology, Renji Hospital, Shanghai Jiao Tong University School of Medicine (SJTUSM), Shanghai, China; 6 Liver Disease Center, Shanghai Public Health Clinical Center, Fudan University, Shanghai, China; 7 Department of Respiratory Medicine, Shanghai Public Health Clinical Center, Fudan University, Shanghai, China

**Keywords:** COVID-19, Type I interferons, Neutralizing antibodies, IFN-α2, IFN-ω

## Abstract

**Objective:**

We sought to explore the prevalence of type I interferon-neutralizing antibodies in a Chinese cohort and its clinical implications during the Omicron variant wave of SARS-CoV-2.

**Methods:**

Type I interferon (IFN) autoantibodies possessing neutralizing capabilities were identified using luciferase assays. The capacity of the autoantibodies for in vitro interference with antiviral activity of IFN was assessed by using a SARS-CoV-2 replicon system. An analysis of the demographic and clinical profiles of patients exhibiting neutralizing antibodies was also conducted.

**Results:**

In this cohort, 11.8% of severe/critical cases exhibited the existence of type I IFN-neutralizing antibodies, specifically targeting IFN-α2, IFN-ω, or both, with an elderly male patient tendency. Notably, these antibodies exerted a pronounced inhibitory effect on the antiviral activity of IFN against SARS-CoV-2 under controlled in vitro conditions. Furthermore, a noteworthy correlation was discerned between the presence of these neutralizing antibodies and critical clinical parameters, including C-reactive protein (CRP) levels, D-dimer levels, and lymphocyte counts.

**Conclusion:**

The presence of type I IFN-neutralizing antibodies is a pervasive risk factor for severe/critical COVID-19 in the Chinese population.

**Supplementary Information:**

The online version contains supplementary material available at 10.1007/s10875-024-01683-z.

## Introduction

The recent declaration by the World Health Organization (WHO) that COVID-19 no longer represents a public health emergency represents a significant turning point in worldwide efforts to address the ongoing pandemic. Nevertheless, it is imperative to underscore that the enduring consequences of COVID-19 remain an essential issue in the realm of public health. With approximately 770 million confirmed infections and a tragic death toll nearing 7 million individuals worldwide, the virus continues to exert a profound impact. Although most individuals infected with SARS-CoV-2 exhibit minor symptoms, approximately 10% of patients develop severe or critical manifestations of the disease, often necessitating life-saving interventions such as mechanical ventilation [1]. Numerous factors have been identified as potential contributors to the severity of COVID-19. These factors include advanced age, obesity (defined as a body mass index exceeding 23), pregnancy, and comorbidities such as chronic kidney disease, diabetes, hypertension, asthma, and malignancies. [2–7] However, these factors alone do not comprehensively explain the determinants of life-threatening COVID-19 cases. Consequently, recent research has shifted its focus to host genetic and immune factors, which have become pivotal areas of investigation.

The innate immune response plays a pivotal role in the host's reaction to viral infection, with the interferon (IFN) system serving as a critical component. Activation of the IFN system subsequently induces downstream inflammatory factors and antiviral proteins, directly inhibiting viral replication and mediating subsequent immune responses. Prior research has suggested heightened susceptibility to life-threatening infections in individuals with monogenic inborn errors of immunity (IEIs) and those possessing autoantibodies neutralizing specific cytokines. Notably, patients with inborn errors of IFN-γ immunity exhibit vulnerability to weakly virulent mycobacteria (Mendelian susceptibility to mycobacterial disease [MSMD]) and the more virulent *Mycobacterium tuberculosis *[8]. Importantly, JL Casanova and colleagues identified single-gene mutations associated with type I interferon signaling pathways (TLR3, IRF7, IRF3, TICAM1/TRIF, UNC93B1, TBK1, IFNAR1, and IFNAR2) in COVID-19 patients that may result in life-threatening outcomes in life-threatening cases of COVID-19 pneumonia [9]. Since the 1980s, researchers have for the first time identified type I interferon-neutralizing antibodies in patients with disseminated herpes zoster syndrome, followed by subsequent discoveries of similar antibodies in individuals affected by various severe viral diseases. These antibodies have been detected in patients with critical influenza pneumonia (5%), critical Middle East respiratory syndrome (20%), severe adverse reactions to yellow fever as a live attenuated vaccine (30%), and in patients with West Nile virus encephalitis (40%) [10–14]. In particular, research has revealed the presence of neutralizing antibodies against IFN-α2 and/or IFN-ω in approximately 13% of individuals with critical COVID-19 pneumonia. Only approximately 1% tested positive for neutralizing antibodies specific to IFN-β. In contrast, these autoantibodies are rarely found among no-life-threatening COVID-19 patients [15–23]. Notably, the existing research lacks data from the Chinese population. In this study, we sought to address this knowledge gap by investigating the prevalence of IFN-α2 and/or IFN-ω neutralizing antibodies and their correlation with clinical outcomes in Chinese COVID-19 patients during an outbreak of the SARS-CoV-2 Omicron variant in a multicenter cohort in Shanghai, China.

## Materials and Methods

### Patients

This retrospective study involved the collection of serum samples from COVID-19 patients infected with the Omicron variant at the Shanghai Public Health Clinical Center and the Shanghai Sixth People's Hospital during the outbreak period from March to July 2022. A total of 184 COVID-19 patients were included in the study. The inclusion criteria were based strictly on the "Scheme for Diagnosis and Treatment of 2019 Novel Coronavirus Pneumonia (The 9th Trial Edition)" and WHO standards for clinical classification [24]. Patients meeting any of the following criteria were classified as having life-threatening COVID-19 pneumonia: a respiratory rate exceeding 30 breaths per minute, resting oxygen saturation less than 93%, an oxygenation index of ≤ 300 mmHg or lung infiltration exceeding 50%, and requiring mechanical ventilation. There were 85 patients in this cohort. The remaining patients were classified as having the moderate type, which is characterized by exhibiting clinical symptoms and radiological features indicative of COVID-19 pneumonia, or the mild type, which is defined by relatively mild clinical symptoms and no radiological evidence of pneumonia. There were 50 patients in the moderate-type group and 49 patients in the mild-type group. Additionally, plasma samples from 100 adults younger than 60 years were collected before the outbreak period as controls. Plasma or serum samples were collected from all patients during the acute phase of the illness.

### Autoantibody Function Assessment

Reporter luciferase activity was assessed to determine the neutralizing activity of autoantibodies against IFN-α2 and IFN-ω. In brief, HEK293T cells were transfected with plasmids containing the human ISRE promoter-controlled firefly luciferase gene with the pGL4.45 backbone and constitutively expressing Renilla luciferase. The cells were cultured in DMEM supplemented with 10% serum/plasma from healthy controls or patients. The cells were then stimulated with either 10 ng/ml or 100 pg/ml interferon-alpha 2 (IFN-α2) or interferon-omega (IFN-ω) at 37 °C for 16 h. Each sample was tested once. Subsequently, the cells were subjected to lysis at ambient temperature for 20 min. Quantification of luciferase levels was performed utilizing the Dual-Luciferase Reporter Assay System (Promega, catalog number E1980). The activity values of firefly luciferase were standardized by normalization to the those of Renilla luciferase. The samples were deemed to possess neutralizing activity if luciferase induction, when normalized to Renilla luciferase activity, was less than 15% of the mean control value.

### Assessment of Antiviral Activity of IFN Against SARS-CoV-2 Replicon in the Presence of Patient Serum

To assess the capacity of the autoantibodies in the patient serum for in vitro interference with the antiviral activity of IFN, we used a SARS-CoV-2 replicon system based on the SARS-CoV-2 nCoV-SH01 strain, the original strain of SARS-CoV-2 [25]. This replicon contains a secreted Gaussian luciferase (sGluc) gene encoded in the viral subgenomic mRNA, which served as a reporter gene and is devoid of structural proteins such as spike (S), membrane (M), and envelope (E). Huh7 cells were seeded in 96-well plates at a density of 4.5 × 10^4^ cells per well. These cells were exposed to a mixture of IFN-α2/IFN-ω and patient serum for 4 h. Subsequently, they were co-transfected with wild-type (WT) or replication-defective SAA mutant replicon RNA and N mRNA using the TransIT-mRNA Transfection Kit (Mirus) without changing the medium, following the manufacturer's instructions. At 48 h post-transfection, the supernatants were taken and combined with an equivalent amount of 2 × passive lysis buffer (Promega). The luciferase activity was measured using the Renilla luciferase substrate (Promega) following the methods provided by the manufacturer.

### Data Analysis and Statistics

We employed R version 4.2.2 or GraphPad Prism version 9.4.1 for statistical analyses. Continuous variables are reported as medians and standard deviations (SDs), whereas binary variables are presented as event counts and standard deviations. Categorical variable comparisons were carried out using Fisher's exact test; Mann‒Whitney and Kolmogorov‒Smirnov tests were utilized for comparisons of continuous variables. To evaluate the influence of autoantibodies that neutralize type I interferons on the severity of COVID-19, we employed Firth's bias-corrected logistic regression, as implemented in the "logistf" R package, to calculate odds ratios (ORs) and P values. Before this analysis, propensity score matching was conducted to address potential confounding factors due to baseline characteristics.

## Results

### Demographic and Clinical Characteristics of the Study Cohort

In our cohort, a total of 184 COVID-19 patients were included, consisting of 85 individuals with life-threatening COVID-19, 50 with moderate symptoms, and 49 classified as mild cases. Significant disparities were observed among life-threatening, moderate, and mild COVID-19 patients in our study cohort. Age was significantly different across severity levels, with median ages of 82, 64, and 31 for life-threatening, moderate, and mild, respectively (*p* < 0.001). There were significant differences in the prevalence of comorbidities, including hypertension, diabetes, heart disease, renal disease, malignant tumor, and neuropsychopathy, as well as in the usage of antiviral agents, glucocorticoids, anticoagulation, vaccination status, and both invasive and non-invasive mechanical ventilation (*p* < 0.001). Other symptoms such as fever and cough also showed significant differences across severity levels. Moreover, the life-threatening group had more extended hospitalization and a greater mortality rate than the other groups. All patient groups' detailed demographic and clinical features are listed in Table 1.

**Table 1:** Demographics and clinical characteristics of patients on admission

	Overall	Life-threatening COVID-19	Μoderate	Μild	P
Demographics					
N	184	85	50	49	
Age (median [IQR])	70.50 [40.00, 86.00]	82.00 [71.00, 89.00]	64.00 [42.00, 84.50]	31.00 [22.00, 45.00]	< 0.001
0–59	70 (38.0)	6 (7.1)	20 (40.0)	44 (89.8)	
60–69	21 (11.4)	13 (15.3)	6 (12.0)	2 (4.1)	
70–79	26 (14.1)	17 (20.0)	8 (16.0)	1 (2.0)	
80-	67 (36.4)	49 (57.6)	16 (32.0)	2 (4.1)	
Sex (male(%))	117 (63.6)	52 (61.2)	32 (64.0)	33 (67.3)	0.773
Comorbidities					
Hypertension (%)	72 (39.1)	51 (60.0)	19 (38.0)	2 (4.1)	< 0.001
Diabetes (%)	40 (21.7)	27 (31.8)	12 (24.0)	1 (2.0)	< 0.001
Heart disease (%)	45 (24.5)	33 (38.8)	10 (20.0)	2 (4.1)	< 0.001
Renal disease (%)	34 (18.5)	29 (34.1)	5 (10.0)	0 (0.0)	< 0.001
Malignant tumor (%)	22 (12.0)	16 (18.8)	5 (10.0)	1 (2.0)	0.014
Neuropsychopathy (%)	64 (34.8)	45 (52.9)	15 (30.0)	4 (8.2)	< 0.001
Symptoms at admission					
Fever (%)	54 (29.3)	34 (40.0)	10 (20.0)	10 (20.4)	0.013
Cough (%)	73 (39.7)	42 (49.4)	16 (32.0)	15 (30.6)	0.043
Treatment					
Glucocorticoids (%)	59 (32.1)	53 (62.4)	5 (10.0)	1 (2.0)	< 0.001
Anticoagulation (%)	91 (49.5)	74 (87.1)	16 (32.0)	1 (2.0)	< 0.001
Antiviral agent ( (Nirmatrelvir/ritonavir) %)	66 (35.9)	59 (69.4)	7 (14.0)	0 (0.0)	< 0.001
Vaccination	86 (46.7)	17 (20.0)	22 (44.0)	47 (95.9)	< 0.001
Days of hospitalization (median [IQR])	13.00 [8.00, 23.25]	23.00 [15.00, 31.00]	8.00 [4.25, 14.00]	9.00 [5.00, 11.00]	< 0.001
O2.requirements					
Nasal cannula (%)	20 (10.9)	10 (11.8)	9 (18.0)	1 (2.0)	0.036
High flow reservoir mask (%)	18 (9.8)	18 (21.2)	0 (0.0)	0 (0.0)	< 0.001
Invasive or non-invasive mechanical ventilation (%)	58 (31.5)	56 (65.9)	2 (4.0)	0 (0.0)	< 0.001
Total death (%)	23 (12.5)	22 (25.9)	1 (2.0)	0 (0.0)	< 0.001

Data are reported as N, *N* (%), or median (interquartile range). A Fisher test was used to analyze the effect of dichotomous variables, and a Mann–Whitney test was used for continuous variables. A *P* value < 0.05 was considered to indicate statistical significance.

### Enrichment of Neutralizing Autoantibodies Against IFN-α2 and IFN-ω within Life-threatening COVID-19 Patients

**Fig. 1:**
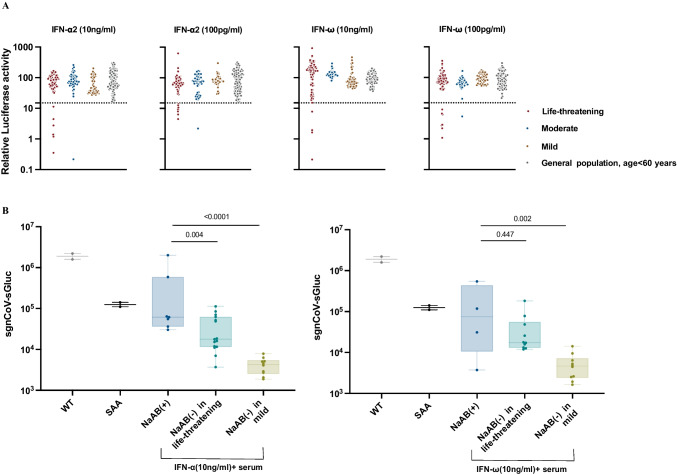
Prevalence of IFN-α2- and/or IFN-ω-neutralizing antibodies in life-threatening COVID-19 patients and verification of their inhibitory effect on interferon-mediated antiviral activity in vitro. (**A**) The results for neutralization of 10 ng/ml or 100 pg/ml IFN-α2 and 10 ng/ml or 100 pg/ml IFN-ω in the presence of plasma 1/10 from patients with life-threatening COVID-19 (*n* = 85), moderate patients (*n* = 50), mild group (*n* = 49) and a group of general population (*n* = 100) are shown. The assessments were performed using 293 T cells co-transfected with a firefly luciferase reporter gene under the control of an interferon-stimulated response element (ISRE) and an internal control vector encoding Renilla luciferase. Stimulation by IFN was measured by relative luciferase activity, which was calculated by normalizing firefly luciferase activity to Renilla luciferase activity. (**B**) Huh7 cells were seeded in 96-well plates and incubated at 37℃ overnight. IFNα/IFNω was diluted in media containing 2% FBS and then incubated with patients’ plasma in a total 50ul mixture at 37℃ for 15 min. Then, the cells were replaced with the mixture and incubated at 37℃ for another 4 h. The replicon RNA was transfected into the cells, and the luciferase activity in the supernatant was determined at 48 h post-transfection. Statistical analysis was performed using one-way ANOVA

The presence of neutralizing autoantibodies against type I interferons (IFNs) was evaluated in blood samples obtained from the previously mentioned COVID-19 cohort. For this assessment, we conducted an evaluation in which we introduced a firefly luciferase reporter gene regulated by the ISRE element and a control plasmid encoding Renilla luciferase into human embryonic kidney (HEK) 293 T cells. Subsequently, we stimulated these cells with either IFN-α2 or IFN-ω, which are individual human recombinant type I interferons, under two conditions: in the absence or presence of a 1:10 dilution of plasma obtained from patients or controls. The interferon response of the transfected 293 T cells was subsequently evaluated by assessing induction of firefly luciferase activity after normalization to that of Renilla luciferase. We thus identified that among the 85 severe/critical COVID-19 patients, 10 patients (11.8%, 95% CI [6.5–20.3]) had plasma-neutralizing antibodies against IFN-α2 and/or IFN-ω. In contrast, only 2 out of 50 cases in the moderate subgroup displayed such autoantibodies. Importantly, none of the 49 samples from the mild group or the general population samples of 100 adults younger than 60 years demonstrated detectable IFN-α2 or IFN-ω neutralizing antibodies (Fig. 1A). In addition, among 85 life-threatening COVID-19 patients, 17 individuals had received two or more doses of the inactivated SARS-CoV-2 vaccine. Among these vaccinated individuals, two patients (12%) tested serologically positive for neutralizing antibodies against interferon-α2 and interferon-ω. One patient exhibited serum autoantibodies (auto-Abs) neutralizing both IFN-α2 and IFN-ω at concentrations of 10 ng/ml and 100 pg/ml, respectively, whereas the serum from the other patient neutralized IFN-α2 at 10 ng/ml and 100 pg/ml, but only neutralized IFN-ω at 100 pg/ml. Thus, neutralizing antibodies against type I interferons appear to be associated with an increased risk of breakthrough hypoxemic COVID-19 pneumonia among the Chinese population previously vaccinated with the inactivated SAR-CoV-2 vaccine.

Patients with autoantibodies that neutralize type I interferons can be further classified according to the neutralization spectrum. Among the 10 individuals identified in the life-threatening group, 30.0% (3/10) exhibited serum neutralization activity against both IFN-α2 (10 ng/ml) and IFN-ω (10 ng/ml), which was absent in the moderate/mild group. Additionally, 90% (9/10) and 80% (8/10) exhibited neutralizing antibodies against IFN-α2 (100 pg/ml) and IFN-ω (100 pg/ml) alone, respectively. In contrast, only a single case was observed in the moderate group in both categories. We also observed a higher prevalence of these autoantibodies in aged patients. Among patients aged 60 and above, 7.0% exhibited neutralizing antibodies against IFN-α2 (10 ng/ml), 3.5% against IFN-ω (10 ng/ml), 2.6% against both IFN-α2 and IFN-ω (10 ng/ml), and 7.9% against either IFN-α2 or IFN-ω (10 ng/ml). In contrast, patients under 60 years old did not show neutralizing antibodies against IFN-α2 or IFN-ω (10 ng/ml). Detailed information is provided in Table 2.

**Table 2:** Prevalence of neutralizing antibodies to type I IFNs in 184 COVID-19 patients, stratified by disease severity, age, or sex

10 ng/ml						100 pg/ml			
Severity	No. of patients	IFN-α2	IFN-ω	IFN-α2 and IFN-ω	IFN-α2 or IFN-ω	IFN-α2	IFN-ω	IFN-α2 and IFN-ω	IFN-α2 or IFN-ω
Life-threatening	85	7(8.2%[4.0–16.0])	4(4.7%[1.8–11.5])	3(3.5%[1.0–9.9])	8(9.4%[4.8–17.5])	9(10.6%[5.7–18.9])	8(9.4%[4.8–17.5])	8(9.4%[4.8–17.5])	10(11.8%[6.5–20.3])
Moderate	50	1(2.0%[0.1–10.5])	0(0.0%)	0(0.0%)	1(2.0%[0.1–10.5])	1(2.0%[0.1–10.5])	1(2.0%[0.1–10.5])	0(0.0%)	2(4.0%[0.7–13.5])
Mild	49	0(0.0%)	0(0.0%)	0(0.0%)	0(0.0%)	0(0.0%)	0(0.0%)	0(0.0%)	0(0.0%)
Total	184	8(4.3%[2.2–8.3])	4(2.2%[0.8–5.5])	3(1.6%[0.4–4.7])	9(4.9%[2.6–9.0])	10(5.4%[3.0–9.7])	9(4.9%[2.6–9.0])	8(4.3%[2.2–8.3])	12(6.5%[3.8–11.1])
Age	No. of patients	IFN-α2	IFN-ω	IFN-α2 and IFN-ω	IFN-α2 or IFN-ω	IFN-α2	IFN-ω	IFN-α2 and IFN-ω	IFN-α2 or IFN-ω
0–59	70	0(0.0%)	0(0.0%)	0(0.0%)	0(0.0%)	0(0.0%)	1(1.4%[0.1–7.7])	0(0.0%)	1(1.4%[0.1–7.7])
60–69	21	1(4.8%[0.2–22.7])	0(0.0%)	0(0.0%)	1(4.8%[0.2–22.7])	1(4.8%[0.2–22.7])	1(4.8%[0.2–22.7])	1(4.8%[0.2–22.7])	1(4.8%[0.2–22.7])
70–79	26	1(3.8%[0.2–18.9])	1(3.8%[0.2–18.9])	1(3.8%[0.2–18.9])	1(3.8%[0.2–18.9])	1(3.8%[0.2–18.9])	1(3.8%[0.2–18.9])	1(3.8%[0.2–18.9])	1(3.8%[0.2–18.9])
80-	67	6(9.0%[4.2–18.2])	3(4.5%[1.2–12.4])	2(3.0%[0.5–10.2])	7(10.4%[5.2–20.0])	8(11.9%[6.2–21.8])	6(9.0%[4.2–18.2])	6(9.0%[4.2–18.2])	9(13.4%[7.2–23.6])
Sex	No. of patients	IFN-α2	IFN-ω	IFN-α2 and IFN-ω	IFN-α2 or IFN-ω	IFN-α2	IFN-ω	IFN-α2 and IFN-ω	IFN-α2 or IFN-ω
Female	68	2(2.9%[0.5–10.1])	0(0.0%)	0(0.0%)	2(2.9%[0.5–10.1])	3(4.4%[1.2–12.2])	2(2.9%[0.5–10.1])	2(2.9%[0.5–10.1])	3(4.4%[1.2–12.2])
Male	116	6(5.2%[2.4–10.8])	4(3.4%[1.3–8.5])	3(2.6%[0.7–7.3])	7(6.0%[3.0–12.0])	7(6.0%[3.0–11.9])	7(6.0%[3.0–11.9])	6(5.2%[2.4–10.8])	9(7.8%[4.1–14.1])

### The Autoantibodies Antagonize the in vitro Inhibitory Effect of IFN-α2 and IFN-ω on SARS-CoV-2 Replicon

It has been well documented that coronaviruses including SARS-CoV-2 are highly sensitive to IFN [26]. To explore the physiologic relevance of the presence of IFN autoantibodies, we next investigated whether the autoantibodies present in patient sera antagonize the anti-SARS-CoV-2 activity of IFN-α2 and IFN-ω. To this end, we used an IFN-sensitive, non-infectious replicon system based on the original SARS-CoV-2 strain [25] as described in material and methods. This analysis compared the impact of IFN on suppression of the SARS-CoV-2 replicon in serum samples obtained from COVID-19 patients. Specifically, the samples were categorized into three groups: Group 1 consisted of patients with neutralizing antibodies to IFN-α2 or IFN-ω and was named the NaAb( +) group, Group 2 and 3 are both NaAb(-) groups that lack such autoantibodies, included patients with life-threatening and no-life-threatening COVID-19, respectively. As expected, the highest reporter gene expression was observed in the Group 1 serum samples, consistent with the effective blockade of autoantibody-matched type I IFN action. Interestingly, Group 2 sera allowed for a level of reporter gene expression that fell between Group 1 and Group 3 sera. When assayed against IFN-α2, group 1 sera showed significantly higher antagonizing activity compared to group 2 sera. However, the differences between the two groups did not reach statistical significance in IFN-ω-neutralizing activity. This lack of significance could be attributed to the limited sample size of group 1 (Fig. 1B). Nevertheless, the IFN-α2 /IFN-ω-neutralizing antibodies presented in the patient sera were capable of antagonizing the inhibitory effects of physiological levels of IFN-α2 and IFN-ω on the SARS-CoV-2 replicon in human cells, emphasis their in vivo efficacy in counteracting the antiviral effects of type I interferons during SARS-CoV-2 infection.

### Age, Sex, and Selective Hematological and Serological Parameters are Associated with the Prevalence of Neutralizing Antibodies to Type I Interferons in COVID-19 Patients

**Fig. 2:**
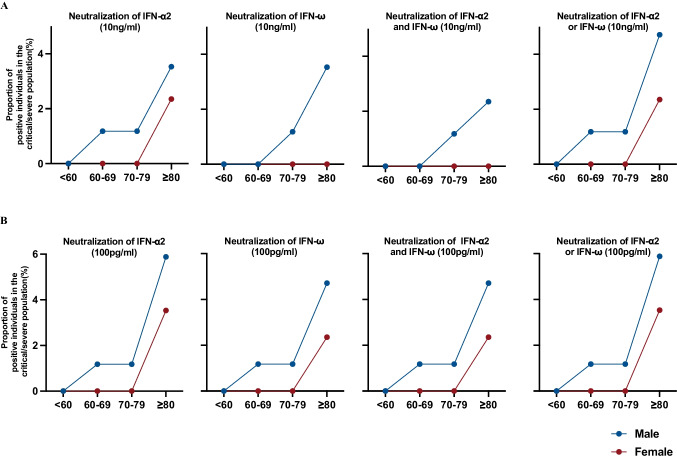
Autoantibodies neutralizing IFN-α2 and/or IFN-ω are more common in males and elderly individuals among severe or critical COVID-19 patients. Percentages of men versus women among severe or critical COVID-19 patients positive for neutralizing auto Abs (in 1:10 dilution of plasma) against (A) IFN-α2 only, IFN-ω only, IFN-α2 and IFN-ω, IFN-α2 and/or IFN-ω at 10 ng/ml; (B) IFN-α2 only, IFN-ω only, IFN-α2 and IFN-ω, IFN-α2 and/or IFN-ω at 100 pg/ml

We next sought to identify patient characteristics related to the prevalence of neutralizing antibodies against type I interferons. We analyzed the correlation of autoantibodies with age and sex. The median age of patients harboring autoantibodies was 83.50 [79.50, 89.75] years, and 75% (9/12) were male. Age distribution analysis revealed a link between age and the occurrence of autoantibodies. Specifically, among patients aged 60 years and older with life-threatening COVID-19, 11.8% (95% CI: 6.5–20.3) exhibited neutralizing antibodies against IFN-α2, and 9.4% (95% CI: 4.8–17.5) had neutralizing antibodies against IFN-ω; in contrast, among patients aged younger than 60 years, none had IFN-α2 or IFN-ω neutralizing antibodies (Fig. 2). Sex distribution analyses indicated that approximately 7.1% (95% CI: 3.3–14.6) and 8.2% (95% CI: 4.0–16.0) of the male patients had circulating autoantibodies neutralizing IFN-α2 and/or IFN-ω at concentrations of 10 ng/ml and 100 pg/ml, respectively; these two numbers decreased to 2.4% and 3.5%, respectively, among the female counterparts (Fig. 2). Thus, our data suggest that development of interferon-neutralizing autoantibodies in the Chinese population is age and sex dependent.

**Fig. 3:**
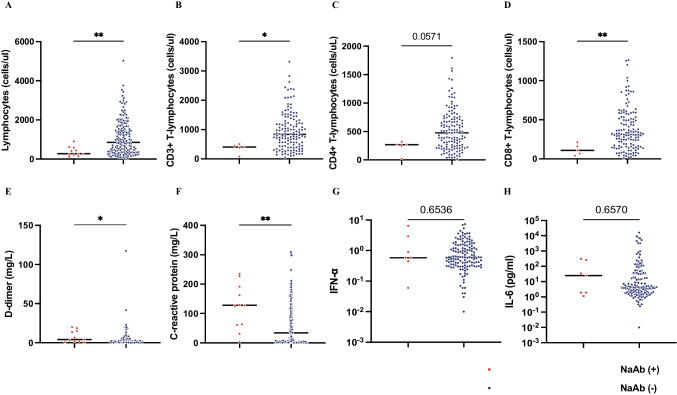
Correlations between clinical laboratory parameters and type I interferon-neutralizing antibodies in COVID-19 patients. Comparison between IFN-α and/or IFN-ω autoantibody-negative and IFN-ω autoantibody-positive patient groups according to the following extreme values: (**A**) absolute lymphocyte count, (**B**) absolute CD3 + T lymphocyte count, (**C**) absolute CD4 + T lymphocyte count, (**D**) absolute CD8 + T lymphocyte count, (**E**) C-reactive protein (CRP), (**F**) D-dimer, (**G**) IFNα, and (**H**) IL-6. Statistical analysis was performed using the Mann‒Whitney U test. *P value < 0.05 or **P value < 0.01

We also explored relationships between clinical phenotypes, treatment regimens, and interferon-neutralizing autoantibodies. No significant differences were observed in comorbidities, initial symptoms at admission, or oxygen requirements between patients with and without such autoantibodies (Table S1). Comprehensive comparison of laboratory test results revealed that the presence of interferon-neutralizing autoantibodies correlated with a notable decrease in lymphocyte count and increased D-dimer and C-reactive protein levels (Fig. 3A-F). Patients with neutralizing antibodies did not exhibit a significant decrease in serum IFN-α or IL-6 levels (Fig. 3G-H), and there was no No substantial association was observed between interferon-neutralizing autoantibodies and cytokine levels, as depicted in Figure S1.

### Type I Interferon-neutralizing Antibodies Contribute to Adverse COVID-19 Disease Outcomes

**Fig. 4:**
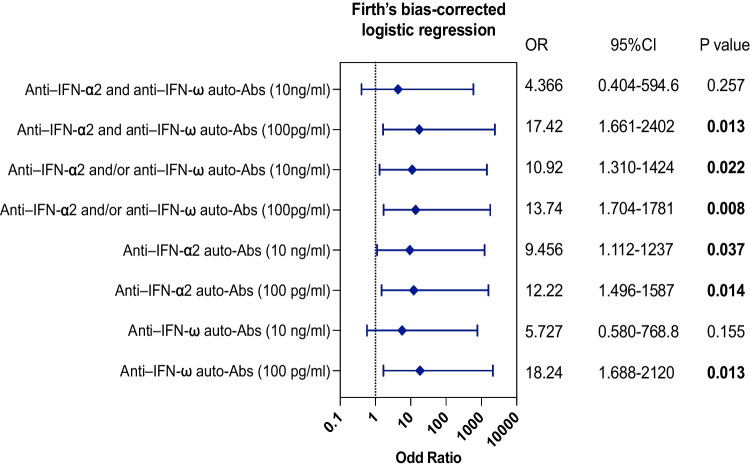
Assessment of type I IFN autoantibodies as a potential risk factor for life-threatening COVID-19. Odds ratios (ORs) and *P* values were estimated using Firth’s bias-corrected logistic regression, with adjustment for age and sex

Finally, we analyzed the association between serum antibody levels against type I interferon and unfavorable outcomes related to COVID-19. To account for potential confounding factors, such as age and sex, we employed propensity score matching (PSM). Our study population exhibited an increased odds ratio (OR) for developing life-threatening COVID-19 in individuals with neutralizing antibodies against IFN-α2 and IFN-ω. This association was particularly evident in individuals with autoantibodies that were capable of antagonizing IFN-ω at a concentration of only 100 pg/ml (OR = 18.24 *P* = 0.013) and in those who possessed autoantibodies that effectively neutralized both IFN-α2 and IFN-ω at the same concentration (OR = 17.42,* P* = 0.013) (Fig. 4). Unexpectedly, we were unable to detect a significant correlation between a higher level of interferon-neutralizing antibodies (neutralizing IFN-α2 and IFN-ω at 10 ng/ml) and an increased risk of life-threatening COVID-19. We believe that the limited sample size might be the possible reason for this discrepancy, and we anticipate that enrolling a larger cohort in future investigations will help clarify this aspect.

## Discussion

In this study, we focused on a cohort that was infected and hospitalized during the Omicron wave in China/Shanghai. This cohort differs from cohorts in previous studies in two ways. Firstly, their hospitalization was exclusively caused by infection with the Omicron variant BA.2.2, which has milder clinical symptoms compared to earlier strains. During the BA.2.2 wave, 85.4% of the infected population were categorized as having mild symptoms, 13.4% as moderate, and only 2.76% as severe/critical [27–29]. Secondly, as part of strong non-pharmaceutical public health intervention (NPI) measures, a large proportion of the cohort had received two or three doses of wild-type SARS-CoV-2 inactivated vaccines before being exposed to BA.2.2. Using this cohort, we aimed to determine whether autoantibodies neutralizing type I IFNs present an independent risk factor for the severity of COVID-19 during the Omicron wave in China/Shanghai. Among the subset of severe/critical COVID-19 patients, 11.8% tested positive for neutralizing antibodies against IFN-α2 and/or IFN-ω at a concentration of 100 pg/ml. The presence of these neutralizing antibodies was found to correlate with a higher likelihood of mortality, as reflected by the fact that 17.4% of deceased COVID-19 patients harbored these antibodies. These findings are closely aligned with a large-scale study by "Human Genetic Effort," which reported prevalence rates for neutralizing antibodies against IFN-α and/or IFN-ω in different cohorts ranging from 13.7% to 18% [16]. Similar prevalence rates have been confirmed in other studies conducted in various regions worldwide, including Spain (10.6% prevalence rate), the United States (9% prevalence rate), Russia (10.5% prevalence rate), Japan (13.2% prevalence rate), Italy (11.9% prevalence rate), and the Netherlands (10% prevalence rate) [18–20, 23, 30–35]. We also found that life-threatening COVID-19 patients possessing neutralizing antibodies against IFN-α2 and/or IFN-ω tended to be elderly males, a pattern consistent with observations made with other patient populations [16, 19, 22, 23, 36, 37].

Notably, we employed an in vitro assay utilizing the SARS-CoV-2 replicon system to validate that the serum from most patients harboring IFN-α and/or IFN-ω autoantibodies is capable of neutralizing the SARS-CoV-2 inhibitory activity of the respective type I interferon(s).Interestingly, when assayed against IFNω stimulation, we did not observe a significant statistical difference in the replicon inhibition between the life-threatening antibody-positive and the life-threatening antibody-negative groups, despite a discernible trend. We speculate that this may be attributed to the relatively small sample size of the IFNω antibody-positive group. Subsequently, we will include more samples in our follow-up work to validate our findings.

We examined correlations between the presence of type I interferon-neutralizing antibodies and routine clinical parameters. Although we did not observe significant differences in IFN-α, IL-6, or other cytokine levels between patients with and without naAbs, this may be related to the fact that we did not detect a peak in cytokine elevation during the early stages of infection. However, our findings showed that individuals who possessed neutralizing antibodies against type I interferons exhibited notably elevated C-reactive protein (CRP) and D-dimer levels and decreased lymphocyte counts. C-reactive protein (CRP) is a commonly employed indicator of inflammation, and D-dimer is a well-accepted parameter for assessing coagulation status. Extensive clinical studies have confirmed that increased CRP and D-dimer levels and decreased lymphocyte counts are reliable indicators of severe/critical COVID-19 and poor outcomes [7]. Indeed, these indicators are now integrated into clinical risk scoring systems for COVID-19 mortality, assisting clinicians in assessing patient conditions and providing care. Although further investigations are needed to determine how type I interferon-neutralizing antibodies contribute to the elevated IFN-reactive protein (CRP) and D-dimer levels in COVID-19 patients, the likely mechanism is as follows: the presence of such neutralizing antibodies undermines the ability of host innate immunity to restrain SARS-CoV-2 infection, leading to uncontrolled spread of the virus and consequently more extensive tissue damage and elevated inflammation. Interestingly, we observed a noticeable effect of glucocorticoid treatment on reducing CRP levels, supporting its usage for clinical improvement. In our cohort, 32.1% (59 out of 184) of patients received glucocorticoid treatment during clinical management, with a usage rate of 62.4% for life-threatening cases. Out of the 8 patients who tested positive for neutralizing antibodies to IFN-α2/IFN-ω, corticosteroid treatment reduced C-reactive protein (CRP) levels in all cases except one. In light of this, glucocorticoid treatment may be considered for these patients. Glucocorticoids can not only inhibit the production of inflammatory cytokines and chemokines but also induce proteins with anti-inflammatory activity [38].

An important clinical implication of our study is that, for Chinese populations, screening for type I interferon-neutralizing antibodies at the time of hospital admission may provide an early warning indicator regarding whether patients are likely to experience progression to a severe or critical condition. These findings corroborate previous studies conducted on individuals of other ethnic backgrounds. Patients with type I interferon-neutralizing antibodies should be managed promptly and benefit from early intervention, such as administration of anti-SARS-CoV-2 medications, use of virus-neutralizing antibodies, and administration of inhibitors or immunoglobulins targeting anti-inflammatory cytokines [39]. When convalescent plasma therapy is used, the donor must also be screened for type I interferon-neutralizing antibodies, and any individual whose plasma is positive for such antibodies should be excluded [40]. Previous studies have provided evidence that although anti-IFN-α and/or IFN-ω autoantibodies are commonly observed, the presence of anti-IFN-β autoantibodies in patients with COVID-19 is infrequently reported [16]. Although we did not analyze the presence of anti-IFN-β autoantibodies in our study cohorts, it is highly likely that these antibodies also occur less frequently in the Chinese population than anti-IFN-α and/or IFN-ω autoantibodies. Therefore, administering IFN-β at an early stage of SARS-CoV-2 infection may be considered a viable option for effectively inhibiting viral replication [16].

A growing body of evidence supports a genetic basis for the propensity to produce autoantibodies. An exemplary case was demonstrated in patients with autoimmune polyendocrine syndrome type 1 (APS1), which is caused by autosomal recessive mutations in the AIRE gene. APS1 patients are prone to producing neutralizing autoantibodies against type I interferon, increasing their risk of contracting life-threatening pneumonia upon COVID-19 infection [41–44]. Neutralizing autoantibodies against the IL-17 cytokine were also identified in APS1 patients and are considered the basis for the development of chronic mucocutaneous candidiasis [45, 46]. These findings unveiled a unified genetic mechanism underlying the production of disease-causing autoantibodies. Additional support for the existence of a genetic predisposition toward the production of autoantibodies came from a recent study. The study revealed that individuals with NF-κB deficiency are prone to developing type I interferon-neutralizing autoantibodies [47]. Further investigation of the etiology underlying production of type I interferon-neutralizing autoantibodies, including their age- and sex-dependency, may lead to new opportunities for developing more effective therapies for not only severe/critical COVID-19 but also other viral infections that are tightly controlled by type I IFN signaling.

We acknowledge that the size of the study group limits our study and that the results of some of our correlation analyses may need to be validated in the future with an expanded cohort. There is also a limitation in our functional characterization of the IFN-neutralizing autoantibodies. Although we have successfully demonstrated in human cells that these autoantibodies are capable of blocking the inhibitory activity of matched type I IFN on a replicon derived from a SARS-CoV-2 strain highly related to the original Wuhan prototype, it is desirable to directly assess their effects on the physiologically more relevant Omicron infection using authentic viruses. However, conducting the related experiments in a BSL-3 laboratory is currently not feasible for us. We are interested in conducting them in future studies. We also acknowledge several limitations associated with our replicon assay. Only neutralizing activities against a high dose of type I IFN (10 ng/mL) were assessed and no positive control (e.g. purified anti-IFN neutralizing antibody) was included. In addition, substantial variations within subgroups could be attributed to confounding factors in the plasma, which can be addressed by using IgG purified from the sera. Nevertheless, our study first explored type I interferon-neutralizing antibodies and their roles in COVID-19 diseases in a Chinese population. One distinguishing factor of our research, in comparison to prior studies conducted in different nations, is use of cohorts comprising individuals with the Omicron variant, which is overall less pathogenic than wild-type SARS-CoV-2 and other variants, with the assessments of type I interferon-neutralizing antibodies being performed before progression to life-threatening conditions. Thus, our findings provide new lines of evidence supporting the generality of the notion that type I interferon-neutralizing autoantibodies are preexisting risk factors for life-threatening COVID-19; thus, avoiding the adverse effects of these antibodies should be generally considered when treating COVID-19 patients.

### Supplementary Information

Below is the link to the electronic supplementary material.

Supplementary file1 (EPS 2961 KB)

Supplementary file2 (DOCX 138 KB)

## Data Availability

The data supporting this study's findings are available from the corresponding author upon reasonable request.
