# Supplementary material for: Prevalence of Neutralizing Autoantibodies Against Type I Interferon in a Multicenter Cohort of Severe or Critical COVID-19 Cases in Shanghai
Source: J Clin Immunol. 2024 Mar 10;44(3):80. doi: 10.1007/s10875-024-01683-z (PMC10925575; doi:10.1007/s10875-024-01683-z)
Supplement: Supplementary file 2 — Supplementary file2 (DOCX 138 KB) [file 10875_2024_1683_MOESM2_ESM.docx]

**Table S1 Comparing demographic and clinical characteristics of patients with and without type I interferon-neutralizing antibodies**

D Data are reported as N, N (%), or median (interquartile range). A Fisher test was used to analyze the effect of dichotomous variables,

and a Mann–Whitney test was used for continuous variables. A *P* value < 0.05 was considered to indicate statistical significance.
